# Dynamic changes of bone microarchitecture and volumetric mineral density assessed by HR-pQCT in patients with cervical cancer after concurrent chemoradiotherapy: a prospective study

**DOI:** 10.1186/s40364-025-00754-6

**Published:** 2025-03-18

**Authors:** Weishi Cheng, Yijun Wu, Jing Shen, Hui Guan, Li Zhang, Hongnan Zhen, Yinjie Tao, Weibo Xia, Zhikai Liu, Fuquan Zhang

**Affiliations:** 1 Department of Radiation Oncology, Peking Union Medical College Hospital, Chinese Academy of Medical Sciences and Peking Union Medical College, Beijing, China; 2 Department of Hematology, West China Hospital, Sichuan University, Chengdu, Sichuan China; 3 Key Laboratory of Endocrinology of National Health Commission, Department of Endocrinology, Peking Union Medical College Hospital, Chinese Academy of Medical Sciences and Peking Union Medical College, Beijing, China; 4 Peking Union Medical College, Chinese Academy of Medical Sciences, Beijing, China

**Keywords:** High-resolution peripheral quantitative computed tomography, Cervical cancer, Bone mineral density, Bone microstructure, Chemoradiotherapy

## Abstract

Bone changes in patients undergoing pelvic radiotherapy remain unclear. This study initially utilized high-resolution peripheral quantitative computed tomography (HR-pQCT) to assess the dynamic changes in bone microarchitecture and volumetric bone mineral density (BMD) in patients with cervical cancer before and after concurrent chemoradiotherapy. This prospective, observational study included patients with squamous carcinoma of the cervix scheduled for concurrent chemoradiotherapy. Patients underwent HR-pQCT, dual-energy X-ray absorptiometry (DXA) and laboratory tests before chemoradiotherapy, and at three and six months post-chemoradiotherapy. DXA, serving as the clinical standard for measuring BMD, was employed alongside HR-pQCT to provide complementary insights into bone micro-changes. The primary endpoint comprised changes in total (Tt.vBMD), trabecular (Tb.vBMD) and cortical (Ct.vBMD) volumetric BMD at the distal radius and tibia between pre-chemoradiotherapy and 6 months post-chemoradiotherapy. A total of 21 patients were enrolled, and one patient chose to withdraw (median age: 54.5 years). Tt.vBMD significantly decreased three months (distal radius: -1.65%, *P* = 0.008; distal tibia: -2.4%, *P* < 0.001) and six months (distal radius: -3.03%, *P* = 0.003; distal tibia: -2.69%, *P* = 0.002) post-chemoradiotherapy compared to baseline. Similarly, Tb.vBMD and Ct.vBMD demonstrated a significant downward trend post-chemoradiotherapy, with mean percent changes at three months of -0.73% and − 1.59% for the distal radius, and − 1.95% and − 1.50% for the distal tibia, respectively. The trends in BMD changes measured by DXA align with those observed using HR-pQCT. Regarding the laboratory tests, estradiol levels significantly decreased post-chemoradiotherapy, while follicle stimulating hormone and luteinizing hormone levels significantly increased. The results found that concurrent chemoradiotherapy was associated with the changes in bone volume, microstructure and BMD, especially in BMD three months post-chemoradiotherapy. Most of the bone micro-changes had not reverted by six months. This study explored the feasibility of early fracture risk identification post-chemoradiotherapy, aiding physicians in taking timely measures to improve prognosis.

**Supplementary Information:**

The online version contains supplementary material available at 10.1186/s40364-025-00754-6.

**To the editor**.

Pelvic insufficiency fractures (PIFs) are a common complication following pelvic radiotherapy, a critical treatment modality in cervical cancer for enhancing survival [1, 2]. Sapienza et al. [2] performed a meta-analysis of 21 studies, including 3929 female patients who underwent pelvic radiotherapy, and found that 14% of patients developed pelvic fractures after radiotherapy. A small-scale prospective study demonstrated that 89% of patients experienced PIFs within a year after receiving pelvic radiotherapy [3]. The absence of established preventive or therapeutic methods emphasizes the importance of early fracture risk assessment in these patients. Fracture risk is strongly correlated with bone mineral density (BMD) [4], and currently, dual-energy X-ray absorptiometry (DXA) is the clinical standard for measuring BMD. However, its reliance on two-dimensional projection makes DXA susceptible to interference from surrounding tissue densities, potentially leading to underestimated fracture risks [5, 6]. Advances in high-resolution peripheral quantitative computed tomography (HR-pQCT) provide a non-invasive and more accurate assessment of bone microstructure and volumetric BMD by reconstructing the three-dimensional bone structure [7, 8]. HR-pQCT has enabled independent assessment of trabecular and cortical bones through quantitative bone microstructure parameters, effectively addressing the limitations of DXA and facilitating the early identification of fracture risk. To date, none of studies has been reported regarding the bone micro-changes in patients undergoing pelvic radiotherapy. This prospective study initially utilized HR-pQCT to assess the dynamic changes in bone microarchitecture and volumetric BMD in patients with cervical cancer before and after concurrent chemoradiotherapy.

This prospective, observational study enrolled patients with squamous carcinoma of the cervix scheduled for concurrent chemoradiotherapy between September 2022 and April 2024. Patients underwent HR-pQCT, DXA and laboratory tests related to bone metabolism and sex hormones before the first chemoradiotherapy session, and at 3-month and 6-month intervals following the completion of concurrent chemoradiotherapy. The primary endpoint comprised changes in total (Tt.vBMD), trabecular (Tb.vBMD) and cortical (Ct.vBMD) volumetric BMD at the distal radius and tibia between pre-chemoradiotherapy and 6 months post-chemoradiotherapy. The study design and profile are elaborated in Fig. 1A and B, respectively, while the inclusion and exclusion criteria, treatment, measurement and statistical analysis are detailed in Supplementary Material 2: Methods. The study protocol was approved by the Ethics Committee of Peking Union Medical College Hospital (No. ZS-3266) and followed the Declaration of Helsinki and Good Clinical Practice guidelines. Written informed consent was obtained from all participants.

**Fig. 1:**
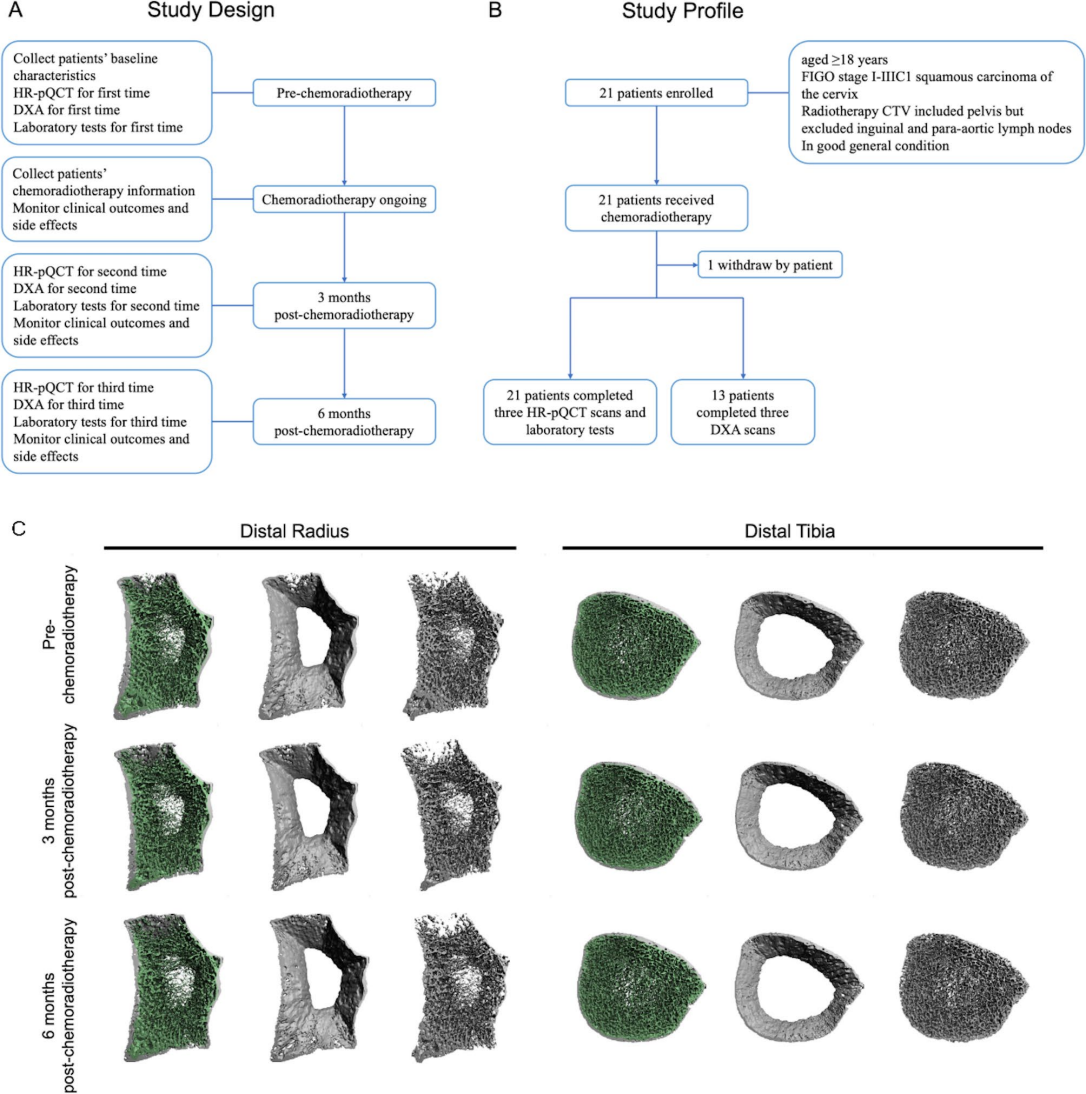
Flowchart of the study and representative 3-dimensional bone microarchitecture images of distal radius and tibia. **A**. Study design. **B**. Study profile. **C**. Representative 3-dimensional bone microarchitecture images of distal radius and tibia from one patient in the cohort. There were subtle differences between pre-chemoradiotherapy and post-chemoradiotherapy. CTV: clinical target volume; DXA: dual energy X-ray absorptiometry; FIGO: International Federation of Gynecology and Obstetrics staging system; HR-pQCT: high-resolution peripheral quantitative

A total of 21 patients were enrolled, and one patient chose to withdraw (median age: 54.5 years). The remaining 20 patients successfully underwent three HR-pQCT scans and corresponding laboratory tests, while 13 patients completed three DXA examinations as scheduled. Clinical characteristics of the patients are summarized in Supplementary Material 1: Table S1. And the three HR-pQCT scan images of one patient in this cohort are shown in Fig. 1C. Tt.vBMD significantly decreased three months (distal radius: *P* = 0.008; distal tibia: *P* < 0.001) and six months (distal radius: *P* = 0.003; distal tibia: *P* = 0.002) post-chemoradiotherapy compared to baseline (Table 1). Similarly, Tb.vBMD and Ct.vBMD exhibited a significant downward trend post-chemoradiotherapy (Table 1). Significant changes were also noted in certain bone microstructure and geometric parameters at the distal radius and tibia, including cortical thickness (Ct.Th) and cortical bone area (Ct.Ar) (Table 1). The mean percent changes in the three BMD indices obtained by HR-pQCT pre- and post-chemoradiotherapy are shown in Supplementary Material 1: Figure S1, visually illustrating the downward trend in patients’ BMD levels, with these changes persisting at 6 months post-chemoradiotherapy. Additionally, the trends in BMD changes measured by DXA align with those observed using HR-pQCT. The BMD and Z-score levels of the lumbar spine, femoral neck and total hip significantly decreased at both three and six months post-chemoradiotherapy, compared to baseline (Table 1). Regarding the laboratory tests, estradiol levels significantly decreased at six months post-chemoradiotherapy compared to baseline, while follicle stimulating hormone and luteinizing hormone levels significantly increased (Supplementary Material 1: Table S2). Additionally, laboratory results generally positively or negatively correlated with HR-pQCT BMD parameters at the distal radius and tibia, the correlations between them are depicted in Supplementary Material 1: Figure S2 and S3. Correlations between dose-volume indexes of bone (total, spongy, and cortical) and percent changes in HR-pQCT BMD parameters post-chemoradiotherapy are shown in Supplementary Material 1: Table S3 and S4.

**Table 1:** Comparisons of bone parameters obtained by HR-pQCT and DXA between pre-chemoradiotherapy and post-chemoradiotherapy

Bone parameters	Pre-chemoradiotherapy	3 months post-chemoradiotherapy	6 months post-chemoradiotherapy	*P*-value^a^	*P*-value^b^	*P*-value^c^
HR-pQCT: distal radius						
Tt.vBMD (mgHA/cm^3^)	294.4 (259.7, 365.7)	288.4 (246.0, 363.9)	284.4 (231.6, 352.5)	**0.008**	**0.003**	**0.009**
Tb.vBMD (mgHA/cm^3^)	116.6 (87.63, 120.1)	112.0 (84.53, 127.6)	112.0 (82.00, 123.3)	0.086	**0.014**	**0.004**
Ct.vBMD (mgHA/cm^3^)	932.2 (893.5, 978.8)	922.6 (875.6, 981.8)	918.9 (868.4, 975.5)	0.121	0.121	0.573
Ct.Pm (mm)	66.95 (61.80, 72.33)	67.20 (61.98, 71.83)	67.05 (61.98, 70.23)	0.616	1.000	0.812
Ct.Ar (mm^2^)	58.40 (50.60, 66.60)	58.60 (49.78, 66.38)	56.25 (49.83, 63.98)	**0.046**	**0.002**	**0.004**
Tb.Ar (mm^2^)	196.0 (163.7, 230.3)	196.7 (164.6, 231.8)	198.5 (166.5, 232.8)	**0.033**	**0.002**	**0.007**
Tb.BV/TV (%)	0.173 (0.121, 0.179)	0.167 (0.113, 0.183)	0.165 (0.113, 0.186)	0.601	0.177	0.106
Tb.N (mm^− 1^)	1.110 (1.018, 1.372)	1.112 (0.997, 1.349)	1.073 (0.991, 1.346)	0.422	0.117	0.240
Tb.Th (mm)	0.221 (0.208, 0.231)	0.223 (0.210, 0.232)	0.220 (0.207, 0.232)	0.757	0.517	0.442
Tb.Sp (mm)	0.869 (0.683, 0.960)	0.879 (0.669, 0.980)	0.873 (0.688, 0.972)	0.490	0.322	0.313
Ct.Th (mm)	1.059 (0.843, 1.207)	1.053 (0.848, 1.189)	1.032 (0.821, 1.197)	**0.019**	**0.002**	**0.026**
Ct.Po (%)	0.004 (0.002, 0.007)	0.004 (0.002, 0.007)	0.004 (0.003, 0.006)	0.120	0.092	1.000
HR-pQCT: distal tibia						
Tt.vBMD (mgHA/cm^3^)	263.8 (224.5, 289.6)	260.0 (219.1, 283.7)	260.7 (216.9, 280.1)	**< 0.001**	**0.002**	0.150
Tb.vBMD (mgHA/cm^3^)	116.0 (91.90, 142.2)	113.6 (90.28, 138.8)	115.4(90.65, 139.4)	**0.003**	0.052	0.542
Ct.vBMD (mgHA/cm^3^)	910.3 (850.6, 995.7)	894.8 (837.0, 977.0)	891.3 (834.9, 970.9)	**< 0.001**	**0.001**	0.073
Ct.Pm (mm)	100.2 (95.95, 103.0)	100.4 (96.08, 103.2)	100.2 (96.03, 103.3)	**0.005**	**0.002**	0.744
Ct.Ar (mm^2^)	116.1 (95.00, 131.6)	116.5 (91.90, 128.5)	116.1 (91.25, 129.5)	**0.005**	**0.006**	0.247
Tb.Ar (mm^2^)	563.7 (501.2, 600.8)	566.4 (502.9, 603.2)	566.6 (504.9, 601.3)	**0.004**	**0.005**	0.260
Tb.BV/TV (%)	0.182 (0.152, 0.215)	0.180 (0.150, 0.212)	0.181 (0.145, 0.215)	**0.003**	**0.027**	0.728
Tb.N (mm^− 1^)	1.090 (0.979, 1.250)	1.099 (0.977, 1.213)	1.090 (0.998, 1.216)	0.444	0.556	0.794
Tb.Th (mm)	0.235 (0.223, 0.252)	0.236 (0.221, 0.251)	0.235 (0.222, 0.249)	0.972	0.924	0.623
Tb.Sp (mm)	0.893 (0.784, 0.997)	0.887 (0.790, 1.001)	0.893 (0.782, 0.994)	0.550	0.411	0.862
Ct.Th (mm)	1.376 (1.079, 1.551)	1.344 (1.079, 1.538)	1.373 (1.084, 1.521)	**0.028**	**0.024**	0.197
Ct.Po (%)	0.016 (0.011, 0.033)	0.018 (0.010, 0.031)	0.022 (0.010, 0.030)	0.240	**0.049**	0.272
DXA						
LS BMD (g/cm^3^)	1.110 (0.953, 1.154)	1.053 (0.897, 1.101)	1.044 (0.888, 1.086)	**0.001**	**0.001**	**0.017**
LS Z-score	0.00 (-1.00, 0.40)	-0.60 (-1.30, 0.30)	-0.60 (-1.35, 0.10)	**0.005**	**0.001**	**0.005**
FN BMD (g/cm^3^)	0.875 (0.782, 0.918)	0.838 (0.753, 0.922)	0.828 (0.749, 0.909)	**0.019**	**0.009**	0.345
FN Z-score	0.30 (-0.30, 0.90)	-0.10 (-0.55, 0.55)	-0.30 (-0.85, 0.85)	**0.045**	**0.020**	0.477
TH BMD (g/cm^3^)	0.926 (0.827, 0.980)	0.898 (0.792, 0.969)	0.916 (0.764, 0.964)	**0.003**	**0.006**	0.328
TH Z-score	0.40 (-0.50, 1.15)	-0.10 (-0.75, 0.85)	-0.10 (-1.05, 0.85)	**0.022**	**0.015**	0.259

Bold values indicate statistical significance (*P* < 0.05)

^a^ Comparisons between pre-chemoradiotherapy and 3 months post-chemoradiotherapy

^b^ Comparisons between pre-chemoradiotherapy and 6 months post-chemoradiotherapy

^c^ Comparisons between 3 months post-chemoradiotherapy and 6 months post-chemoradiotherapy

BMD: bone mineral density; Ct.Ar: cortical bone area; Ct.Pm: cortical pore diameter; Ct.Po: cortical porosity; Ct.Th: cortical thickness; Ct.vBMD: cortical volume bone mineral density; DXA: dual energy X-ray absorptiometry; FN: femoral neck; HR-pQCT: high-resolution peripheral quantitative computed tomography; LS: lumbar spine; Tb.Ar: trabecular bone area; Tb.BV/TV: trabecular bone volume to total volume ratio; Tb.N: trabecular number; Tb.Sp: trabecular separation; Tb.Th: trabecular thickness; Tb.vBMD: trabecular volume bone mineral density; TH: total hip; Tt.vBMD: total volume bone mineral density

Bone tissue comprises various cells and matrix components essential for maintaining normal physiological functions. Pelvic irradiation directly alters these components of the pelvic bone and even disrupts the physiological balance of the entire skeletal system, leading to abnormal functions and changes in bone microstructure, thus increasing the risk of fractures [9–11]. Additionally, reduced or absent estrogen levels in patients undergoing pelvic radiotherapy combined with chemotherapy significantly heighten fracture risk [12, 13].

This study was limited by being an observational, single-center, small cohort with a limited number of participants and a short follow-up period. Furthermore, given that concurrent chemoradiotherapy remains a gold standard treatment for cervical cancer, it proves challenging for this study to independently assess the individual impacts of radiotherapy or chemotherapy on bone microstructure and BMD. Due to the lack of a control cohort without high-dose nodal boost, we are unable to assess the correlation between nodal boost to a higher dose and changes in bone micro-changes, which can be potentially designed as a direction for future research. This exploratory study provides a foundation for a randomized controlled trial that will administer calcium supplements to cervical cancer patients undergoing concurrent chemoradiotherapy, aiming to explore further measures to prevent and treat fracture risks.

In this prospective, observational cohort study, we found that concurrent chemoradiotherapy was associated with the changes in bone volume, microstructure and BMD, especially in BMD three months post-chemoradiotherapy. Most of the bone micro-changes had not reverted by six months, underscoring the need for further exploration with extended follow-up periods. The study explored the feasibility of early fracture risk identification post-chemoradiotherapy, aiding physicians in taking timely measures to improve prognosis.

## Electronic supplementary material

Below is the link to the electronic supplementary material.

Supplementary Material 1

Supplementary Material 2

## Data Availability

The datasets used and/or analysed during the current study are available from the corresponding author on reasonable request.

## Abbreviations

BMD: Bone mineral density
Ct.Ar: Cortical bone area
Ct.Pm: Cortical pore diameter
Ct.Po: Cortical porosity
Ct.Th: Cortical thickness
CTV: Clinical target volume
Ct.vBMD: Cortical volume bone mineral density
DXA: Dual energy X-ray absorptiometry
FIGO: International Federation of Gynecology and Obstetrics staging system
FN: Femoral neck
HR-pQCT: High-resolution peripheral quantitative computed tomography
LS: Lumbar spine
PIFs: Pelvic insufficiency fractures
SD: Standard deviation
Tb.Ar: Trabecular bone area
Tb.BV/TV: Trabecular bone volume to total volume ratio
Tb.N: Trabecular number
Tb.Sp: Trabecular separation
Tb.Th: Trabecular thickness
Tb.vBMD: Trabecular volume bone mineral density
TH: Total hip
Tt.vBMD: Total volume bone mineral density
