# Supplementary material for: Dynamic changes of bone microarchitecture and volumetric mineral density assessed by HR-pQCT in patients with cervical cancer after concurrent chemoradiotherapy: a prospective study
Source: Biomark Res. 2025 Mar 18;13:46. doi: 10.1186/s40364-025-00754-6 (PMC11921580; doi:10.1186/s40364-025-00754-6)
Supplement: Supplementary file 1 — Supplementary Material 1 [file 40364_2025_754_MOESM1_ESM.pdf]

Supplementary Material 1

Supplement Figures

Figure S1. Mean percent change in bone mineral density (BMD) parameters obtained by HR-pQCT between pre-chemoradiotherapy and post-chemoradiotherapy. A. Mean percent change in volumetric BMD at the distal radius. B. Mean percent change in volumetric BMD at the distal tibia. The dark blue bars represent BMD parameters 3 months post-chemoradiotherapy, and the light blue bars represent BMD parameters 6 months post-chemoradiotherapy. From left to right in A and B: Tt.vBMD, Tb.vBMD, Ct.vBMD. BMD: bone mineral density; Ct.vBMD: cortical volume bone mineral density; HR-pQCT: high-resolution peripheral quantitative computed tomography; Tb.vBMD: trabecular volume bone mineral density; Tt.vBMD: total volume bone mineral density.

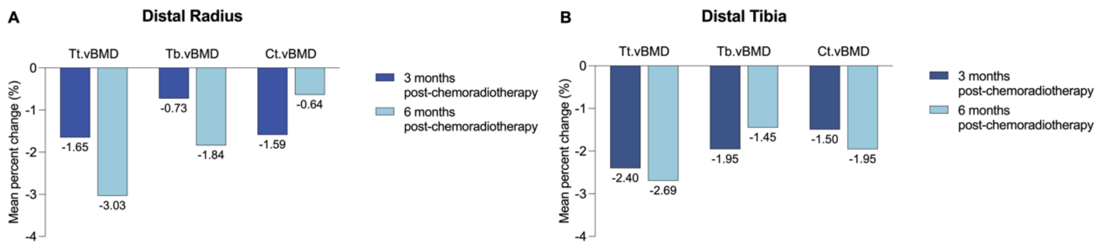

Figure S2. Correlations after clustering between laboratory results and HR-pQCT bone mineral density (BMD) parameters by Spearman analysis at the distal radius. Laboratory results generally positively or negatively correlated with HR-pQCT BMD

parameters. From left to right: pre-radiotherapy, 3 months post-radiotherapy, 6 months post-radiotherapy. ALP: alkaline phosphatase; BMD: bone mineral density; Cr: creatinine; Ct.vBMD: cortical volume bone mineral density; E2: estradiol; FSH: follicle stimulating hormone; HR-pQCT: high-resolution peripheral quantitative computed tomography; iCa: ionized calcium; LH: luteinizing hormone; P: phosphorus; PRL: prolactin; PTH: parathyroid hormone; sCa: serum calcium; sP: serum phosphate; T: testosterone; Tb.vBMD: trabecular volume bone mineral density; TP1NP: total procollagen 1 N-terminal propeptide; Tt.vBMD: total volume bone mineral density; T-25OHD: total 25-hydroxyvitamin D; β-CTX: β-crosslaps of type I collagen. *P < 0.05, **P < 0.01

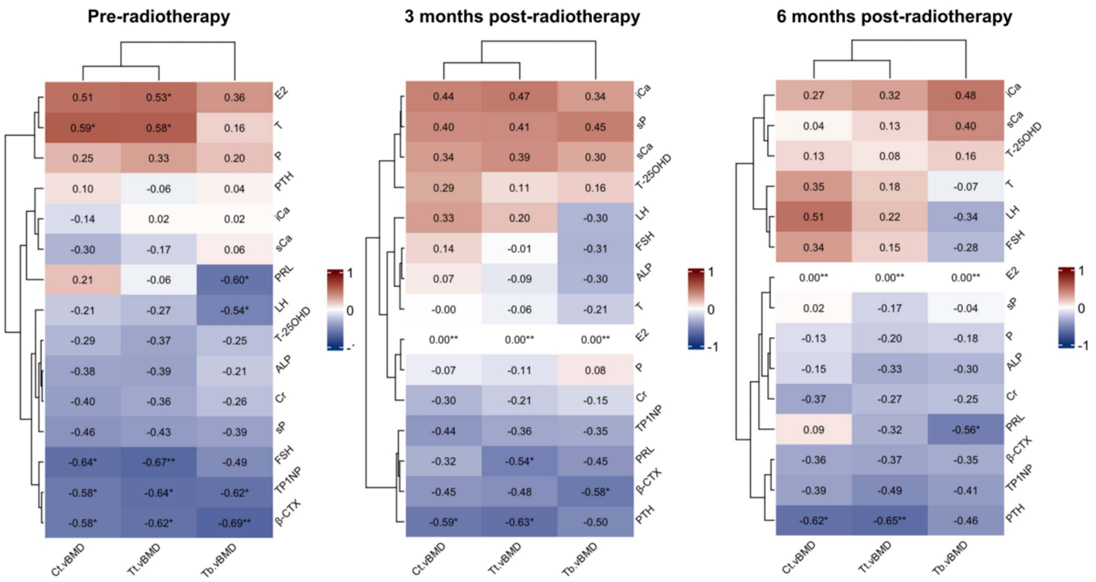

Figure S3. Correlations after clustering between laboratory results and HR-pQCT bone mineral density (BMD) parameters by Spearman analysis at the distal tibia. Laboratory results generally positively or negatively correlated with HR-pQCT BMD parameters. From left to right: pre-radiotherapy, 3 months post-radiotherapy, 6 months post-

radiotherapy. ALP: alkaline phosphatase; BMD: bone mineral density; Cr: creatinine; Ct.vBMD: cortical volume bone mineral density; E2: estradiol; FSH: follicle stimulating hormone; HR-pQCT: high-resolution peripheral quantitative computed tomography; iCa: ionized calcium; LH: luteinizing hormone; P: phosphorus; PRL: prolactin; PTH: parathyroid hormone; sCa: serum calcium; sP: serum phosphate; T: testosterone; Tb.vBMD: trabecular volume bone mineral density; TP1NP: total procollagen 1 N-terminal propeptide; Tt.vBMD: total volume bone mineral density; T-25OHD: total 25-hydroxyvitamin D; β-CTX: β-crosslaps of type I collagen. *P < 0.05, **P < 0.01

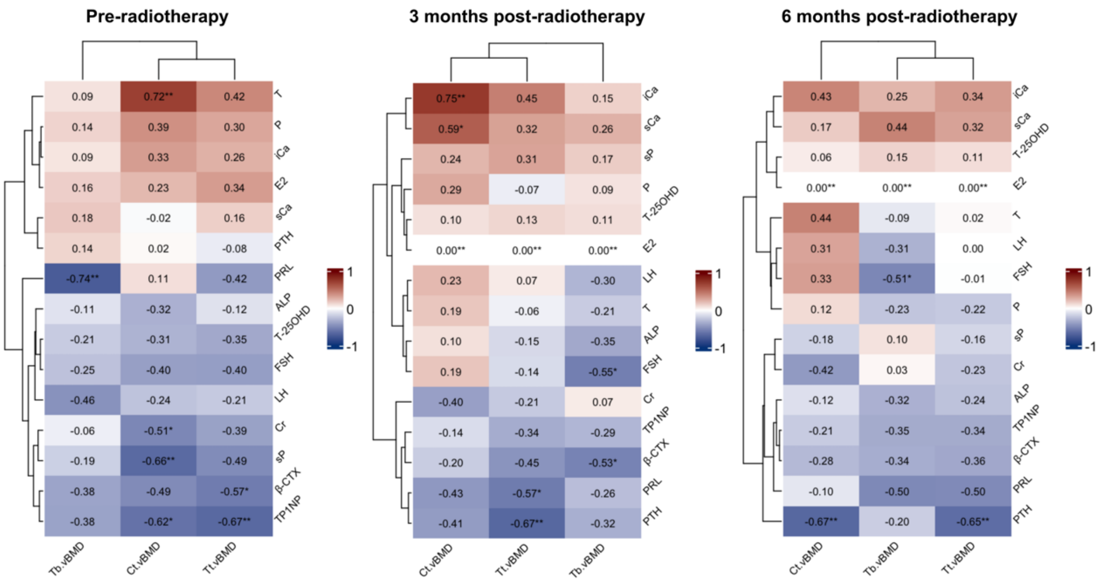

Supplement Tables

Table S1. Clinical characteristics of the patients

Characteristics	All patients (*n*=20)
Age (year), median (range)	54.5 (21-71)
Height (cm), mean ± SD	162.5 ± 6.72
Weight (kg), mean ± SD	64.70 ± 11.91
BMI (kg/m^2), mean ± SD	24.43 ± 3.74
FIGO stage, n (%)	
I	2 (10%)
II	7 (35%)
III	11 (55%)
Menopausal at study entry, n (%)	10 (50%)
Vitamin D deficiency (<20 ng/mL), n (%)	
No	8 (40%)
Yes	12 (60%)

BMI: body mass index; FIGO: International Federation of Gynecology and Obstetrics staging system; SD: standard deviation

Table S2. Comparisons of laboratory results between pre-chemoradiotherapy and post-chemoradiotherapy

Laboratory results	Pre-chemoradiotherapy	3 months post-chemoradiotherapy	6 months post-chemoradiotherapy	*P*-value[a]	*P*-value[b]	*P*-value[c]
iCa (mmol/L)	1.200 (1.160, 1.218)	1.205 (1.170, 1.235)	1.180 (1.160, 1.218)	0.345	0.876	0.298

PTH (pg/ml)	38.85 (30.83, 67.33)	47.70 (29.15, 60.00)	42.80 (27.73, 70.18)	0.918	0.796	0.379
T-25OHD (ng/ml)	15.20 (11.78, 24.68)	11.35 (8.225, 18.03)	17.45 (12.13, 22.00)	0.121	0.496	**0.030**
β-CTX (ng/ml)	0.560 (0.393, 0.830)	0.640 (0.480, 0.885)	0.675 (0.595, 1.033)	0.334	0.074	0.097
TP1NP (ng/ml)	57.85 (38.53, 68.30)	63.75 (45.28. 77.40)	71.60 (51.20, 95.05)	**0.026**	**0.006**	**0.019**
FSH (IU/L)	42.44 (9.390, 63.83)	76.52 (52.56, 88.29)	62.30 (56.34, 101.8)	**0.003**	**0.002**	0.776
LH (IU/L)	26.66 (16.17, 29.56)	31.14 (28.45, 56.03)	34.35 (28.40, 50.81)	**0.011**	**0.036**	0.061
E2 (pg/ml)	7.500 (7.500, 62.00)	7.500 (7.500, 7.500)	7.500 (7.500, 7.500)	**0.028**	**0.028**	1.000
P (ng/ml)	0.335 (0.135, 0.793)	0.250 (0.105, 0.538)	0.185 (0.040, 0.670)	0.086	0.279	0.896
T (ng/ml)	0.280 (0.110, 0.390)	0.180 (0.050, 0.290)	0.190 (0.050, 0.370)	0.093	**0.036**	0.610
PRL (ng/ml)	8.400 (6.400, 11.80)	7.900 (6.300, 9.100)	8.300 (6.400, 11.80)	0.233	0.576	0.118
Serum Ca (mmol/L)	2.390 (2.310, 2.438)	2.395 (2.290, 2.430)	2.330 (2.293, 2.398)	0.955	0.096	0.204
Serum P (mmol/L)	1.330 (1.060, 1.545)	1.305 (1.213, 1.435)	1.240 (1.193, 1.348)	0.955	0.255	**0.025**
ALP (U/L)	90.00 (58.00, 109.0)	72.00 (60.00, 98.00)	74.00 (62.00, 106.0)	0.191	0.421	0.093
Cr (umol/L)	58.50 (51.25, 64.00)	68.00 (56.75, 73.00)	66.00 (55.75, 73.75)	**<0.001**	**0.001**	0.888

Bold values indicate statistical significance (*P* < 0.05)

[a] Comparisons between pre-chemoradiotherapy and 3 months post-chemoradiotherapy

[b] Comparisons between pre-chemoradiotherapy and 6 months post-chemoradiotherapy

[c] Comparisons between 3 months post-chemoradiotherapy and 6 months post-chemoradiotherapy

ALP: alkaline phosphatase; Ca: calcium; Cr: creatinine; E2: estradiol; FSH: follicle stimulating hormone; iCa: ionized calcium; LH: luteinizing hormone; P: progesterone; PRL: prolactin; PTH: parathyroid hormone; serum P: serum phosphate; T: testosterone; TP1NP: total procollagen 1 N-terminal propeptide; T-25OHD: total 25-hydroxyvitamin D; β-CTX: β-crosslaps of type I collagen

Table S3. Correlations between dose-volume indexes of bone (total, spongy, and cortical) and percent changes in HR-pQCT bone mineral density (BMD) parameters post-chemoradiotherapy by Spearman analysis at the distal radius

Dose-volume indexes	3 months post-chemoradiotherapy						6 months post-chemoradiotherapy					
	ΔTt.vBMD (%)		ΔTb.vBMD(%)		ΔCt.vBMD(%)		ΔTt.vBMD (%)		ΔTb.vBMD(%)		ΔCt.vBMD(%)	
	r	*p*	*r*	*p*	*r*	*p*	*r*	*p*	*r*	*p*	*r*	*p*
Total bone												
D_{mean} (Gy)	-0.209	0.391	0.049	0.842	-0.139	0.571	0.030	0.904	0.043	0.861	-0.088	0.721
V_{10} (%)	0.560*	**0.013**	0.369	0.120	0.331	0.166	0.209	0.391	0.406	0.085	0.202	0.407
V_{15} (%)	0.416	0.077	0.312	0.193	0.016	0.949	0.091	0.710	0.284	0.238	0.182	0.455
V_{20} (%)	0.414	0.078	0.465*	**0.045**	-0.023	0.926	0.268	0.267	0.490*	**0.033**	0.204	0.403
V_{30} (%)	0.222	0.361	0.389	0.100	-0.240	0.323	0.244	0.314	0.416	0.077	0.065	0.792
V_{40} (%)	0.319	0.183	0.412	0.079	-0.037	0.881	0.282	0.241	0.426	0.069	0.298	0.215
Spongy bone												
D_{mean} (Gy)	-0.268	0.267	-0.081	0.743	-0.168	0.491	0.025	0.920	-0.083	0.734	-0.109	0.658

V_{10} (%)	0.456*	**0.050**	0.300	0.212	0.150	0.540	0.131	0.593	0.324	0.176	0.119	0.626
V_{15} (%)	0.372	0.117	0.351	0.141	-0.105	0.668	0.214	0.379	0.319	0.182	0.235	0.333
V_{20} (%)	0.316	0.188	0.384	0.104	-0.082	0.737	0.421	0.073	0.343	0.150	0.370	0.119
V_{30} (%)	0.144	0.557	0.180	0.461	-0.112	0.647	0.336	0.159	0.170	0.486	0.219	0.367
V_{40} (%)	0.135	0.581	0.074	0.764	0.061	0.803	0.230	0.344	0.075	0.759	0.239	0.325
Cortical bone												
D_{mean} (Gy)	-0.207	0.395	0.054	0.825	-0.102	0.679	0.004	0.989	0.064	0.794	-0.111	0.652
V_{10} (%)	0.563*	**0.012**	0.385	0.103	0.338	0.157	0.223	0.359	0.414	0.078	0.176	0.472
V_{15} (%)	0.455	0.051	0.329	0.169	0.089	0.718	0.125	0.611	0.358	0.132	0.215	0.377
V_{20} (%)	0.418	0.075	0.451	0.053	-0.046	0.853	0.177	0.468	0.500*	**0.029**	0.133	0.586
V_{30} (%)	0.219	0.369	0.393	0.096	-0.140	0.566	0.122	0.619	0.442	0.058	0.022	0.929
V_{40} (%)	0.316	0.188	0.544*	**0.016**	-0.156	0.523	0.296	0.218	0.542*	**0.016**	0.230	0.344

*Bold values indicate statistical significance ($P < 0.05$)

D_{mean} (Gy) is the mean dose of the structure. V_x (%) refers to the percent volume of bone that receives a radiation dose of at least x Gy.

Ct.vBMD: cortical volume bone mineral density; HR-pQCT: high-resolution peripheral quantitative computed tomography; Tb.vBMD: trabecular volume bone mineral density; Tt.vBMD: total volume bone mineral density

Table S4. Correlations between dose-volume indexes of bone (total, spongy, and cortical) and percent changes in HR-pQCT bone mineral density (BMD) parameters post-chemoradiotherapy by Spearman analysis at the distal tibia.

Dose-	3 months post-chemoradiotherapy	6 months post-chemoradiotherapy

volume indexes	ΔTt.vBMD (%)		ΔTb.vBMD(%)		ΔCt.vBMD(%)		ΔTt.vBMD (%)		ΔTb.vBMD(%)		ΔCt.vBMD(%)	
	r	*p*	*r*	*p*	*r*	*p*	*r*	*p*	*r*	*p*	*r*	*p*
Total bone												
D_{mean} (Gy)	-0.125	0.611	-0.169	0.488	-0.288	0.232	-0.195	0.424	-0.037	0.881	-0.244	0.314
V_{10} (%)	-0.053	0.830	-0.376	0.113	0.039	0.872	0.029	0.906	-0.047	0.847	0.115	0.639
V_{15} (%)	-0.140	0.567	-0.226	0.353	-0.193	0.429	0.002	0.994	0.140	0.567	0	1.000
V_{20} (%)	-0.019	0.937	-0.176	0.470	-0.244	0.314	0.098	0.689	0.114	0.642	-0.033	0.892
V_{30} (%)	-0.022	0.929	-0.200	0.412	-0.184	0.450	0.112	0.647	-0.011	0.963	0.121	0.621
V_{40} (%)	0.002	0.994	-0.183	0.452	-0.242	0.318	0.018	0.943	-0.170	0.486	0.025	0.920
Spongy bone												
D_{mean} (Gy)	-0.151	0.538	-0.208	0.393	-0.235	0.333	-0.237	0.329	-0.139	0.571	-0.170	0.486
V_{10} (%)	-0.205	0.399	-0.462*	**0.046**	-0.104	0.670	-0.160	0.513	-0.219	0.367	0.009	0.972
V_{15} (%)	-0.195	0.424	-0.248	0.305	-0.239	0.325	-0.072	0.770	0.046	0.853	-0.035	0.887
V_{20} (%)	0.091	0.710	-0.104	0.670	-0.039	0.875	0.158	0.519	0.209	0.391	0.205	0.399
V_{30} (%)	0.059	0.811	-0.206	0.397	-0.001	0.997	0.090	0.716	-0.118	0.632	0.300	0.212
V_{40} (%)	-0.011	0.966	-0.233	0.338	-0.116	0.637	-0.012	0.960	-0.267	0.270	0.214	0.379
Cortical bone												
D_{mean} (Gy)	-0.116	0.637	-0.143	0.559	-0.323	0.178	-0.165	0.500	0.009	0.972	-0.293	0.223
V_{10} (%)	-0.061	0.805	-0.342	0.151	0.029	0.906	0.047	0.847	0.025	0.920	0.076	0.756

V_{15} (%)	-0.114	0.642	-0.349	0.143	-0.140	0.569	0.029	0.906	0.071	0.772	0.031	0.901
V_{20} (%)	-0.030	0.904	-0.154	0.528	-0.282	0.241	0.116	0.637	0.146	0.552	-0.079	0.748
V_{30} (%)	-0.054	0.825	-0.227	0.351	-0.304	0.206	0.109	0.657	0.071	0.772	-0.028	0.909
V_{40} (%)	-0.084	0.732	-0.225	0.355	-0.328	0.170	-0.035	0.887	-0.128	0.601	-0.118	0.632

*Bold values indicate statistical significance ($P < 0.05$)

D_{mean} (Gy) is the mean dose of the structure. V_x (%) refers to the percent volume of bone that receives a radiation dose of at least x Gy.

Ct.vBMD: cortical volume bone mineral density; HR-pQCT: high-resolution peripheral quantitative computed tomography; Tb.vBMD: trabecular volume bone mineral density; Tt.vBMD: total volume bone mineral density
