# Supplementary material for: Dynamic changes of bone microarchitecture and volumetric mineral density assessed by HR-pQCT in patients with cervical cancer after concurrent chemoradiotherapy: a prospective study
Source: Biomark Res. 2025 Mar 18;13:46. doi: 10.1186/s40364-025-00754-6 (PMC11921580; doi:10.1186/s40364-025-00754-6)
Supplement: Supplementary file 2 — Supplementary Material 2 [file 40364_2025_754_MOESM2_ESM.pdf]

Supplementary Material 2

Methods

1 Detailed inclusion and exclusion criteria

This study enrolled patients aged ≥18 years with pathologically confirmed International Federation of Gynecology and Obstetrics staging system (FIGO) stage I-IIIC1 squamous carcinoma of the cervix. Eligible patients were scheduled for concurrent chemoradiotherapy, with the radiotherapy clinical target volume (CTV) encompassing the pelvis but excluding the inguinal and para-aortic lymph node areas. Patients were required to be in good general condition, expected to tolerate chemoradiotherapy, and committed to complying with regular follow-ups. Patients with any history of previous radiotherapy or chemotherapy, prior malignancies, previous pelvic surgery, fractures, or diseases affecting bone metabolism, or those who had recently used medications influencing bone metabolism such as parathyroid hormone (PTH), vitamin D, or bisphosphonates were excluded. Patients scheduled chemotherapy from pre-radiotherapy to 6 months post-radiotherapy, or those in poor general condition with a life expectancy of less than one year, were also excluded. Furthermore, patients with severe conditions that could significantly affect study compliance, such as severe infections, cardiovascular or cerebrovascular diseases, mental illnesses, or those deemed unsuitable by the researchers, were not eligible to participate in this study.

2 Treatment

All enrolled patients underwent radical concurrent chemoradiotherapy. Radiotherapy

consisted of external beam radiation and brachytherapy. The CTV for external radiation encompassed the tumor, surrounding high-risk areas, cervix, uterus, parametrium, part of the vagina, and pelvic lymphatic drainage areas (internal iliac, part of external iliac, obturator, presacral, and common iliac), with a prescribed dose of 50.4 Gy in 28 fractions. The gross tumor volume (GTV) for external radiation included the lymph node metastases identified by radiographic evidence, with a prescribed dose of 60.2 Gy in 28 fractions. Brachytherapy was administered using either 2D or 3D techniques, utilizing a Fletcher applicator (with additional interstitial needles if necessary), and employing an Ir-192 source. For 2D brachytherapy, point A served as the reference point to evaluate bladder and rectal doses. For 3D brachytherapy, the high-risk clinical target volume (HR-CTV) served as the prescription dose region to evaluate the doses at the bladder, rectum, sigmoid colon, and small intestine dose to 2 cubic centimeters (D2cc). Concurrently, 4-6 cycles of Cisplatin (DDP) sensitization chemotherapy were given. Dose volume indexes of bone (total, spongy, and cortical) included D_{mean}, V_{10}, V_{15}, V_{20}, V_{30}, V_{40}. D_{mean} (Gy) was the mean dose of the structure. V_x (%) referred to the percent volume of bone that received a radiation dose of at least x Gy.

3 Laboratory tests

The laboratory tests included measurements of serum ionized calcium (iCa), PTH, total 25-hydroxyvitamin D (T-25OHD), β-crosslaps of type I collagen (β-CTX), total procollagen 1 N-terminal propeptide (TP1NP); serum follicle stimulating hormone (FSH), luteinizing hormone (LH), estradiol (E2), progesterone (P), testosterone (T),

prolactin (PRL); and serum calcium (Ca), phosphate (P), alkaline phosphatase (ALP) and creatinine (Cr). All the abovementioned laboratory tests were performed concurrently with the patients' HR-pQCT examinations. Serum iCa levels were determined using the ion-selective electrode method, with measured values corrected for a reference pH of 7.4. T-25OHD, β-CTX and TP1NP levels were determined by an automated Roche electrochemiluminescence system (E601, Roche Diagnostics, Basel, Switzerland). Serum PTH levels were determined by an autoanalyzer (DXI800, Beckman Coulter). FSH, LH, E2, P, T and PRL levels were measured with chemiluminescence (ACS: 180; Automatic Chemiluminescence Systems, Siemens). And serum Ca, P, ALP and Cr levels were determined by an autoanalyzer (AU5800, Beckman Coulter, Brea, CA, USA). Reference ranges were obtained from the central laboratory of Peking Union Medical College Hospital.

4 HR-pQCT

HR-pQCT device (Xtreme CTII; Scanco Medical AG, Bruttisellen, Switzerland) was housed in Department of Endocrinology, Peking Union Medical College Hospital. Operating in standard mode (68 kVp, 1462μA, 100 ms), the device achieved a resolution of 61μm. During measurement, participants were instructed to remain still, and measurements were taken from the non-dominant side's distal radius and tibia, collecting a total of 168 CT slices for each site. Reconstruction of the 3D structure of human bones from the scanning data yielded the following indices: 1) Bone geometric parameters: cortical pore diameter (Ct.Pm), cortical bone area (Ct.Ar), trabecular bone

area (Tb.Ar); 2) BMD indices: total volume bone mineral density (Tt.vBMD), trabecular volume bone mineral density (Tb.vBMD), cortical volume bone mineral density (Ct.vBMD); 3) Bone microstructure indices: trabecular bone volume to total volume ratio (Tb.BV/TV), trabecular number (Tb.N), trabecular thickness (Tb.Th), trabecular separation (Tb.Sp), cortical thickness (Ct.Th), cortical porosity (Ct.Po).

5 DXA

Areal BMD levels of the lumber spine, femoral neck and total hip were measured by DXA (Prodigy Advance, GE Lunar Corporation, Madison, WI, USA) in Department of Radiology, Peking Union Medical College Hospital. Areal BMD levels of the lumber spine were determined by the L1 to L4 areal BMD value. The results were standardized to age-specific Z-scores based on Asian population data.

6 Study endpoints

The primary endpoint of the study comprised changes in volumetric BMD levels of the distal radius and tibia between pre-chemoradiotherapy and 6 months post-chemoradiotherapy including Tt.vBMD, Tb.vBMD and Ct.vBMD. The secondary endpoints included changes in volumetric BMD and bone microstructure parameters of the distal radius and tibia at 3 and 6 months post-chemoradiotherapy, compared to baseline, as assessed by HR-pQCT indices. Changes in areal BMD of the lumbar spine, femoral neck and total hip, measured by DXA before and after chemoradiotherapy, served to validate the HR-pQCT indices. We also analyzed changes in biochemical

measurements related to bone metabolism and sex hormones before and after chemoradiotherapy, to investigate the potential mechanisms through which radiotherapy influenced BMD changes.

7 Statistical Analysis

Statistical analysis of the research data was performed using IBM SPSS 29.0 and R software 4.4.1 (https://www.r-project.org). Numeric variables were represented using medians and quartiles, while categorical variables were represented using frequencies and percentages. The Wilcoxon rank-sum test was employed to evaluate changes in HR-pQCT indices, DXA parameters, and biochemical measurements between pre-chemoradiotherapy and post-chemoradiotherapy. The Spearman analysis was performed to evaluate correlations between laboratory results and HR-pQCT BMD parameters, and correlations between dose-volume indexes of bone (total, spongy, and cortical) around pelvic radiotherapy and percent changes in HR-pQCT BMD parameters post-chemoradiotherapy. Statistical significance was established at P-value < 0.05 for all tests.
